# Enhanced mRNA FISH with compact quantum dots

**DOI:** 10.1038/s41467-018-06740-x

**Published:** 2018-10-26

**Authors:** Yang Liu, Phuong Le, Sung Jun Lim, Liang Ma, Suresh Sarkar, Zhiyuan Han, Stephen J. Murphy, Farhad Kosari, George Vasmatzis, John C. Cheville, Andrew M. Smith

**Affiliations:** 1 Department of Bioengineering, University of Illinois at Urbana-Champaign, Urbana, IL 61801 USA; 2 Micro and Nanotechnology Laboratory, University of Illinois at Urbana-Champaign, Urbana, IL 61801 USA; 3 Intelligent Devices and Systems Research Group, DGIST, Hyeonpung, Daegu, 42988 Republic of Korea; 4 Department of Materials Science and Engineering, University of Illinois at Urbana-Champaign, Urbana, IL 61801 USA; 5 Biomarker Discovery Program, Center of Individualized Medicine, Mayo Clinic, Rochester, MN 55905 USA; 6 Department of Laboratory Medicine and Pathology, Mayo Clinic, Rochester, MN 55905 USA; 7 Carle Illinois College of Medicine, Urbana, IL 61801 USA

## Abstract

Fluorescence in situ hybridization (FISH) is the primary technology used to image and count mRNA in single cells, but applications of the technique are limited by photophysical shortcomings of organic dyes. Inorganic quantum dots (QDs) can overcome these problems but years of development have not yielded viable QD-FISH probes. Here we report that macromolecular size thresholds limit mRNA labeling in cells, and that a new generation of compact QDs produces accurate mRNA counts. Compared with dyes, compact QD probes provide exceptional photostability and more robust transcript quantification due to enhanced brightness. New spectrally engineered QDs also allow quantification of multiple distinct mRNA transcripts at the single-molecule level in individual cells. We expect that QD-FISH will particularly benefit high-resolution gene expression studies in three dimensional biological specimens for which quantification and multiplexing are major challenges.

## Abstract (web summary)

FISH-based techniques to image and count mRNA in single cells can be limited by the photophysical properties of organic dyes. Here the authors develop photostable quantum dot FISH probes for multiplexed imaging.

## Introduction

For a half century, in situ hybridization (ISH) has been used to count, localize, and characterize individual nucleic acids within cells and tissues^1,2^. Originally developed with radioisotopic labels, the advancement of fluorescence ISH (FISH)^3^ led to broad adoption in cytogenetics and clinical diagnostics^4^, today comprising a multi-billion USD industry. Over the past 20 years, FISH has extended to single-RNA analysis^5^, becoming a standardized method for measuring gene expression at the single-cell level, and enabling the discovery of regulatory mechanisms of transcription and translation driven by subcellular localization^6,7^. However signals from organic dye labels used in FISH rapidly deteriorate during photoexcitation, particularly when imaging in three dimensions and under high photon flux needed for super-resolution^8^. In addition, the technique is limited to simultaneous analysis of ~3 RNA targets due to dye emission spectra overlap, unlike high-throughput ex situ techniques like singe-cell whole transcriptome sequencing that simultaneously probe thousands of transcripts from lysed cell extracts. Creative approaches have increased RNA FISH throughput using repeated cycles of labeling, imaging, and label depletion^9^, but the methodologies are laborious and challenging to adopt for non-specialists.

It is widely anticipated that in situ techniques requiring stable, multiplexed probes will substitute dyes with nanocrystalline quantum dots (QDs) due to their extremely stable and intense emission and vastly expanded multiplexing capabilities deriving from narrow emission bands tunable across the ultraviolet, visible, and infrared spectra^10^. But despite concerted efforts, considerable industry investment, and broad use in solution-based assays, QDs have not been widely used in FISH protocols. Presumably this is due to inaccurate labeling resulting from the large sizes (15–35 nm) of commercially available probes^11,12^, which cannot transport into crowded macromolecular environments of fixed cells to densely label targets. To determine the conditions under which QDs can be applied for accurate counting of mRNA transcripts, rigorous quantification must be applied using well-controlled cellular expression systems together with direct comparisons to standardized analytical techniques.

Here we confirm that critical thresholds for cytoplasmic sieving limit RNA FISH and that a new generation of compact and stable QDs can overcome steric hindrance problems to match labeling accuracies of organic dyes. We show that QD-FISH provides improved signal stability, improved fidelity of molecular counting, and the capacity for multiplexed RNA quantification at the single-molecule level.

## Results

### Impact of QD size on mRNA labeling

**Fig. 1:**
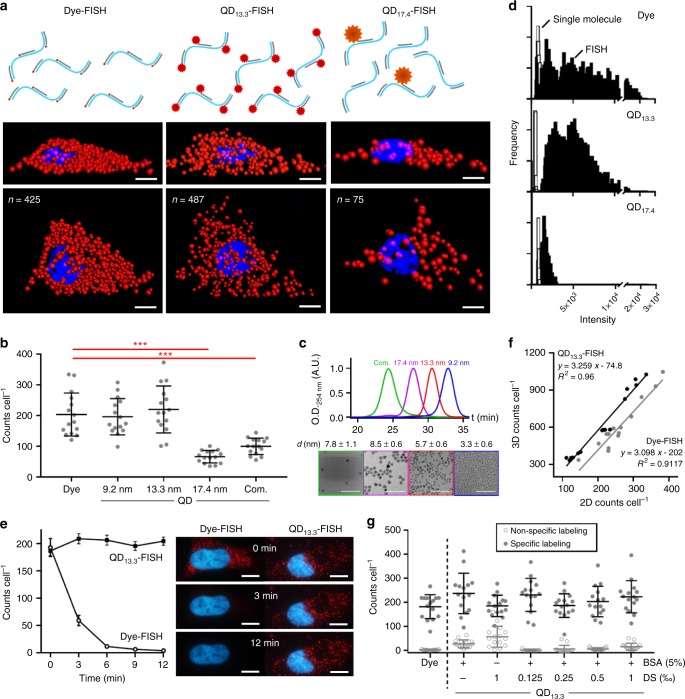
Fluorescence in situ hybridization (FISH) using dye labels or quantum dot (QD) labels with diverse sizes. Data show HeLa cells stained for *glyceraldehyde-3-phosphate dehydrogenase* (*GAPDH*) transcripts. **a** Schematics show RNA target labeling density; representative 3D deconvolved epifluorescence images show cells in two orthogonal orientations, using dyes, small QDs (13.3 nm), or big QDs (17.4 nm). Scale bar = 8 μm. **b** FISH transcript counts (2D) using dyes, custom designed QDs with three hydrodynamic diameters (9.2, 13.3, or 17.4 nm), or commercially available QDs (com.). Asterisks indicate: *p* ≤ 0.05 (*), *p* ≤ 0.01 (**), and *p* ≤ 0.001 (***); Student’s *t*-test. *N* = 15. Comprehensive statistical comparisons are provided in Supplementary Table 2. **c** Gel permeation chromatograms and TEM images (with core size) of the four QDs from panel b. Scale bar = 50 nm. **d** Intensity histograms of FISH spots for dyes, small QDs (13.3 nm), and big QDs (17.4 nm) are shown in black compared with histograms of single-fluorophore intensities in white. **e** FISH counts after different times of laser excitation, comparing stability of QD_13.3_ and dyes, including representative images. Scale bars = 10 μm. *N* = 15. **f** Correlation between FISH counts in 2D and 3D images for QD_13.3_ and dye labels. Comprehensive statistical comparisons are provided in Supplementary Table 1. **g** Impact of customized blocking conditions on specific and nonspecific labeling. Nonspecific labeling counts (2D) for QDs were statistically the same as those of background when applying both 5% bovine serum albumin (BSA) and 0.125‰ dextran sulfate (DS). *N* = 15. All error bars represent s.d

We generated a series of QDs coated with multidentate polymers that allow the total hydrodynamic diameter of the probe to be as small as ~7 nm. These products are stable as off-the-shelf materials for years and are azide-functional for facile conjugation to proteins and nucleic acids through high-precision click-chemistry^13^. Fig. 1a shows representative FISH images of HeLa cells stained for transcripts of *glyceraldehyde-3-phosphate dehydrogenase* (*GAPDH*), comparing ~1 nm organic dyes or QDs with compact (13.3 nm) or large (17.4 nm) hydrodynamic diameters, all using the same oligonucleotide probe sequences based on the Raj et al. multiple labeling method^8^. Similar single-molecule counts are observed for dyes (425) and compact QDs (487), whereas counts for large QDs (75) are much lower. RNA counts per cell for a range of probes are quantified as scatter plots in Fig. 1b. We synthesized these QDs as Hg_*x*_Cd_1−*x*_Se/Cd_*y*_Zn_1−*y*_S core/shell structures with a wide range of diameters of 3.3, 5.7, and 8.7 nm (Fig. 1c), all with emission in the red spectrum (Supplementary Fig. 1), tuned by the core alloy composition parameter *x*^14^. After polymer coating, the hydrodynamic diameters of the respective aqueous QDs were 9.2 nm (QD_9.2_), 13.3 nm (QD_13.3_), and 17.4 nm (QD_17.4_) measured by protein-calibrated gel permeation chromatography (GPC, Fig. 1c). Both QD_9.2_ and QD_13.3_ yielded *GAPDH* mRNA counts that were similar to those of dyes (*p* > 0.05; Student’s *t*-test), whereas counts using QD_17.4_ labels were significantly lower (*p* < 0.001). An alternative large QD variant from a commercial vendor (QD_com_) with dissimilar surface chemistry (PEG-coated amphiphilic polymers) likewise under-labeled RNA targets (*p* < 0.001).

Presumably the majority of *GAPDH* transcripts are located in crowded cytosolic regions inaccessible to QDs larger than 13.3 nm. We specifically chose *GAPDH* as a target due to high expression with distribution throughout the heterogeneous cytoplasm. We observed that the transcripts that could be labeled by QD_17.4_ were also bound to fewer fluorophores compared with QD_13.3_ and dyes. Fig. 1d shows fluorescence intensities per labeled RNA, compared with single fluorophores, showing that RNAs were labeled with a mean of 8.0 dyes, 10 QD_13.3_, and 2.3 QD_17.4_ (Supplementary Table 3). The low labeling density for QD_17.4_ shows that steric hindrance is limiting even for the most accessible RNAs, consistent with reports demonstrating substantially greater obstruction of cytosolic diffusion for ~16 nm pentamers of green fluorescence protein (GFP) than ~11 nm GFP trimers^15^. For the ensuing work below, we exclusively use QD_13.3_.

### Photostability comparisons

Photostability is substantially improved for QD-FISH compared with Dye-FISH. Fig. 1e shows that counts rapidly diminish during photoexcitation of Dye-FISH labeled cells, reducing by ~12% in 30 s and to nearly zero counts in 10 min. This is significant because tens of seconds are needed to acquire a full z-stack for 3D cell imaging. In comparison, QDs exhibit long-term stability with no significant change in *GAPDH* mRNA counts (*p* > 0.05; Student’s *t*-test) after 12 min of excitation, which is consistent with previous results for QD-based stains measured by net intensity^16^. QD-FISH yielded a significantly higher 3D count number compared with Dye-FISH (by 15–30%) for cells with the same 2D counts at a single nuclear focal plane (Fig. 1f). This result likely derives from the rapid decline in dye signal, and is the origin of the lower measured labeling density per transcript for dyes compared with QD_13.3_ (Fig. 1d), which could likely be improved with specialized anti-fade media and dyes optimized for photostability.

### Spot counting fidelity

**Fig. 2:**
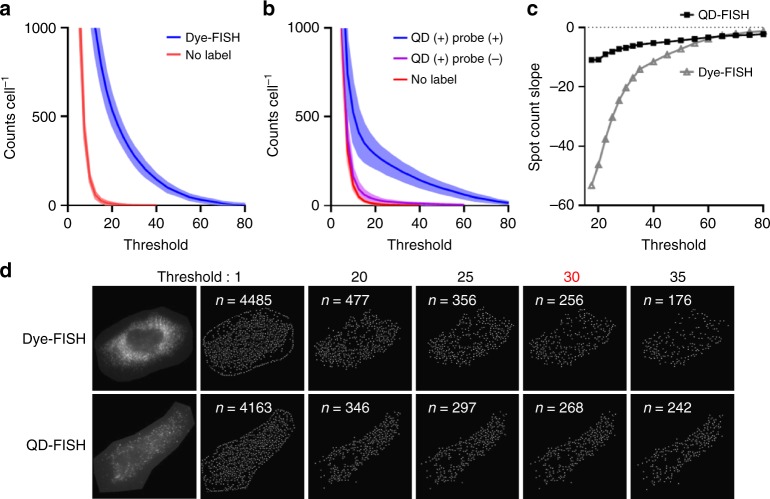
Computational identification of mRNA spots. **a** Spot counts in individual cells using Dye-FISH (blue) or cells without labels (red) for different spot detection thresholds (described further in Methods). Shading indicates s.d. of counts between cells. *N* = 7. **b** Spot counts in individual cells using QD-FISH (blue), cells with QDs added but no probe oligonucleotides (purple), or cells without labels (red), for different detection thresholds. Shading indicates s.d. of counts between cells. *N* = 7. **c** Slopes of positive counts plotted against detection threshold. **d** Representative 2D images of Dye-FISH and QD-FISH are shown on the left, next to calculated images showing the locations of detected spots in white for each of the threshold values indicated above the images. The higher stability of detection for QD-FISH is evident from the similar numbers of detected spots for each of the threshold values spanning 25–35, compared with a wider range of detected spots for Dye-FISH. Inset numbers, *n*, indicate the number of detected spots

The ability to identify puncta corresponding to individual molecules through automated algorithms is also significantly improved with QDs compared with dyes. Numerous image analysis algorithms have been developed to recognize individual fluorescently labeled molecules as diffraction-limited spots, each of which invariably applies a hypothesis test to decide whether a spot should be categorized as positive or negative, typically corresponding to a signal-to-noise threshold for a fit to a two-dimensional Gaussian function. The imposed threshold usually requires ad hoc empirical adjustments through human intervention^8^. Spot counting using a scanning window method with serial image depletion (Multiple Target Tracking algorithm^17^) is shown in Fig. 2a, b for Dye-FISH and QD-FISH, respectively, both using the same probe sequences. The *x*-axis of each plot shows the threshold imposed for spot detection based on the statistical fit of each image spot to a point spread function (described further in Methods). The slope of each positive count curve is plotted in panel c. Compared with Dye-FISH, the curve is much flatter for QD-FISH, indicating a lower sensitivity to threshold selection that is critical for robust automation to eliminate manual selection biases. This outcome derives from the higher brightness of QDs that is far above the spatially variable autofluorescence background (Fig. 2d), whereas the dye channel is highly convolved with autofluorescence and yields a widely varying spot brightness (Fig. 1d).

In the preceding work, we used a 2-step labeling approach in which QDs attached to streptavidin (SAv) label biotinylated nucleic acid probes pre-hybridized to RNA targets. This was necessary for direct comparison of QD with different sizes, as current QD-nucleic acid conjugates have size-dependent valencies. However, the results are statistically the same when comparing this 2-step labeling process using QD_13.3_ with 1-step direct labeling in which the same QDs are conjugated with oligonucleotides before addition to cells (Supplementary Fig. 2–3). The 2-step process also allowed identification of specialized blocking conditions to eliminate nonspecific binding of QDs, which, as solid-phase colloids, have a propensity to adsorb to cellular structures. We used an iterative optimization process (Fig. 1g and Supplementary Fig.s 4–6) to find that both bovine serum albumin (BSA) and polyanions (dextran sulfate; DS) reduce nonspecific binding, and their combination has an additive effect, virtually eliminating nonspecific binding when used together. We attribute the success of this blocking cocktail to the elimination of both denatured hydrophobic domains of proteins (by BSA) and polycationic sites (by DS), both of which can adsorb QDs. However QDs could not be mixed directly with DS due to colloidal aggregation, necessitating sequential blocking. Notably DS requires precise concentration control, having diminishing effect at concentrations greater than 0.125%.

### Validation of labeling accuracy

**Fig. 3:**
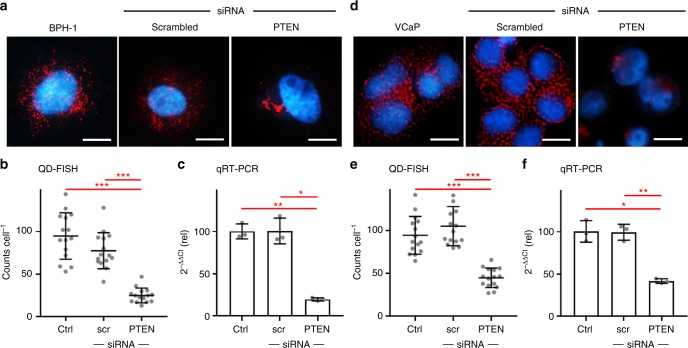
QD-FISH analysis of *phosphatase and tensin homolog* (*PTEN*) transcripts in prostate cancer cell lines. Representative images show BPH-1 cells (**a**) and VCap cells (**d**) with or without treatment by siRNA to knock down *PTEN* expression, or using a scrambled siRNA sequence. Scale bar = 10 μm. Single-cell QD-FISH counts are shown for (**b**) BPH-1 cells and (**e)** VCaP cells, in comparison with transcript measurements by population qRT-PCR for (**c**) BPH-1 cells and (**f**) VCap cells. Significantly reduced mRNA levels are observed for anti-*PTEN* siRNA treatment, with a similar magnitude between QD-FISH and qRT-PCR. Asterisks indicate: *p* ≤ 0.05 (*), *p* ≤ 0.01 (**), and *p* ≤ 0.001 (***); Student’s *t*-test. All error bars represent s.d. *N* = 15 for (**b**) and (**e**), *N* = 3 for (**c**) and (**f**). Comprehensive statistical comparisons are provided in Supplementary Tables 4–7. *PTEN* mRNA FISH probe sequences are shown in Supplementary Table 8

To measure the extent to which exact levels of mRNA can be measured in single cells with QD-FISH, we modulated transcript numbers in cultured cells using short interfering RNA (siRNA). We focus on the transcript of tumor suppressor *phosphatase and tensin homolog* (*PTEN*), a key tumor suppressor gene often deleted in prostate cancer in association with a poor prognosis^18,19^. Representative images for benign prostate hyperplasia (BPH-1) epithelial cells are shown in Fig. 3a, for which an average 75% reduction in *PTEN* RNA counts was measured after siRNA treatment (Fig. 3b), a magnitude similar to that measured at the population level by quantitative reverse transcriptase polymerase chain reaction (qRT-PCR) (Fig. 3c). We performed the same analysis in VCaP prostate cancer cells (Fig. 3d), which are notably much smaller than BPH-1 cells. We again observed a similar magnitude of siRNA-induced transcript knockdown through QD-FISH (Fig. 3e) as that observed at the population level with qRT-PCR (Fig. 3f). This outcome is important because mRNA FISH results are challenging to correlate across cell types with different sizes due to differing degrees of spatial overlap of fluorescent spots^20^.

### Multiplexed QD-FISH

**Fig. 4:**
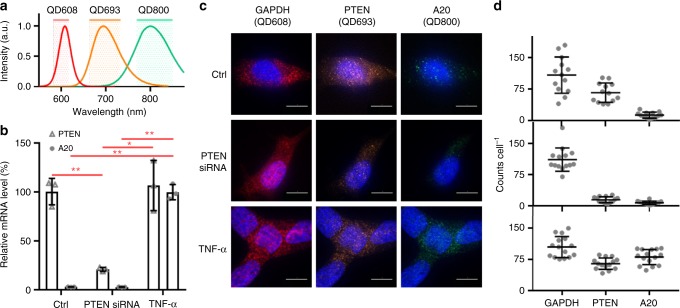
Multiplexed QD-FISH quantification of three transcripts in single LNCaP cells. The three transcripts include *GAPDH*, *PTEN,* and *A20*. **a** Emission spectra of QD608, QD693, and QD800 and corresponding emission bandpass filters used for imaging. **b** Graph shows the expressions of *PTEN* and *A20* transcripts relative to *GAPDH* by qRT-PCR after cells were exposed to either anti-*PTEN* siRNA to knock down *PTEN* expression or TNF-α to induce *A20* expression. Asterisks indicate: *p* ≤ 0.05 (*), *p* ≤ 0.01 (**), and *p* ≤ 0.001 (***); Student’s *t*-test. *N* = 3. **c** Representative QD-FISH images of single cells in each color channel corresponding to bandpass filters shown in panel **a**. QD probes were specific against *GAPDH* (QD608), *PTEN* (QD693) or *A20* (QD800), and cells were treated with or without anti-*PTEN* siRNA or TNF-α. Scale bar = 10 μm. **d** Single-cell QD-FISH results are shown for *GAPDH*, *PTEN* and *A20* transcripts for each of the three experimental conditions corresponding to the images in panel **c**. *N* = 15. All error bars represent s.d. *A20* mRNA FISH probe sequences are shown in Supplementary Table 9

Finally, we validated the ability to use QDs for multiplexed quantification of multiple mRNA sequences at the single-molecule level. We synthesized three QDs (QD608, QD693, and QD800) with spectrally distinct emission bands (Fig. 4a) by tuning the composition of the Hg_*x*_Cd_1−*x*_Se alloy domain, which has little impact on QD size but a substantial impact on electronic bandgap^21^. The three QDs were compact and similar in hydrodynamic size after coating with multidentate polymers by GPC (Supplementary Fig. 7). Each QD was conjugated to oligonucleotides complementary to either *GAPDH* (QD608), *PTEN* (QD693), or *A20* (QD800) mRNA and were then mixed and applied simultaneously to LNCaP prostate cancer cells using the 1-step QD-FISH protocol. Expression levels were modulated by treating the cells with either anti-*PTEN* siRNA to knock down *PTEN* expression, or tumor necrosis factor alpha (TNF-α) to selectively induce *A20* gene expression^22^, each validated by qRT-PCR in Fig. 4b. A representative image from each QD-FISH color channel is shown for individual cells in each experimental group in Fig. 4c, and corresponding transcript counts are shown in panel d. By QD-FISH, *GAPDH* levels were similar in all three experimental groups, whereas *PTEN* transcript level decreased by ~80% with anti-*PTEN* siRNA treatment, and *A20* expression significantly increased with TNF-α treatment. These single-cell results correlated well with the population-level qRT-PCR results.

## Discussion

In this work, we identified a critical size threshold limiting the accuracy of RNA labeling in cells and showed that new QDs with compact sizes can label mRNA targets to yield similar counts as those measured with dyes. The ability to tune QD crystalline size independently from fluorescence emission is a key capability of these materials that allowed us to directly measure the impact of probe size on bioanalytical performance without interference from substantial photophysical mismatch. We anticipate that QD-FISH will drastically  improve single-molecule FISH studies in thick samples for which repetitive excitation leads to rapid signal deterioration and when autofluorescence limits the accuracy of single-molecule identification. These materials should be well suited for studies requiring high-level multiplexing, as the multispectral tunability of QDs is greater any other current fluorescent probe and can exhibit high efficiency emission in the first and second near-infrared spectra where cellular autofluorescence is negligible and detectors have now become affordable^23,24^. New QD engineering approaches have recently become available to widely tune emission wavelengths without changing the size, which is necessary to maintain total minimum dimensions for accurate labeling^14^. The same physicochemical design rules can further be extended to nanoparticle labels such as rare-earth up-conversion materials and responsive plasmonic materials compatible with unique imaging modalities.

## Methods

### Reagents

All chemical reagents were purchased from Sigma-Aldrich or Alfa Aesar unless otherwise specified. QD_com_ was purchased from Thermo-Fisher Scientific as Qdot™ 605 Streptavidin Conjugate.

### QD_9.2_ synthesis

QD_9.2_ with emission at 645 nm and 3.3 nm diameter by electron microscopy was synthesized with a core/shell HgCdSe/CdZnS structure using methods similar to those previously reported^14^. CdSe cores (2.3 nm) were synthesized using a high-temperature injection reaction between cadmium oxide (0.6 mmol), diphenylphosphine selenide (0.2 mmol), and trioctylphosphine selenide (3 mmol) in tetradecylphosphonic acid (1.33 mmol), hexadecylamine (7.1 g), trioctylphosphine (7 mL), and 1-octadecene (ODE; 27.6 mL) at 300 °C for 30 s. After purification, cadmium was partially exchanged with mercury using mercury octanethiolate in oleylamine to yield a HgCdSe core. After purification, a 2.2-monolayer shell of CdZnS was deposited layer-by-layer in 0.8-monolayer increments using cadmium acetate in oleylamine (0.1 M), zinc acetate in oleylamine (0.1 M), and elemental sulfur in ODE (0.1 M) as shell stock solutions. The shell composition comprised 0.8 monolayers of Cd_0.5_Zn_0.5_S, 1.2 monolayers of Cd_0.2_Zn_0.8_S, and 0.2 monolayers of ZnS.

### QD_13.3_ synthesis

QD_13.3_ with emission at 605 nm and 5.7 nm diameter by electron microscopy was synthesized with a core/shell CdSe/CdZnS structure using methods similar to those previously reported^14^. CdSe cores (3.2 nm) were synthesized using a heat-up reaction between cadmium behenate (1 mmol), selenium dioxide (1 mmol), and 1,2-hexadecanediol (1 mmol) in ODE (20 mL) at 230 °C for 15 min. After purification, a 4.7-monolayer shell of CdZnS was deposited using the same methodology as that for QD_9.2_. The shell composition comprised 2.4 monolayers of CdS, 0.8 monolayers of Cd_0.8_Zn_0.2_S, and 1.5 monolayers of ZnS.

### QD_17.4_ synthesis

QD_17.4_ with emission at 680 nm and 8.7 nm diameter by electron microscopy was synthesized with a core/shell CdSe/CdZnS structure. A CdSe core with a first exciton peak at 645 nm was synthesized using a method similar to that for QD_13.3_ with the substitution of cadmium behenate for cadmium myristate. The shell growth process was similar to that used for QD_9.2_ to yield a shell composition of 4.0 monolayers of Cd_0.5_Zn_0.5_S and 1.6 monolayers of ZnS.

### QD800 synthesis

CdSe cores with emission at 549 nm were synthesized in a heat-up reaction mixture of cadmium behenate (0.2 mmol), selenium dioxide (0.2 mmol), and 1,2-hexadecanediol (0.2 mmol) in ODE (5 mL) at 240 °C for 60 min. After purification, cadmium was partially exchanged with mercury to yield a HgCdSe core by mixing the CdSe cores with mercury acetate in oleylamine and chloroform, followed by the addition of octanethiol to quench the reaction. After purification, a shell of CdZnS was deposited using the same methodology as that for QD_9.2_ to yield a shell composition of 2.4 monolayers of CdS, 0.8 monolayers of Cd_0.5_Zn_0.5_S, and 0.8 monolayers of ZnS.

### QD693 synthesis

The synthesis was the same as that used for QD800, except the CdSe core emission wavelength maximum was 558 nm and mercury exchange was performed with mercury octanethiolate in oleylamine to reduce the degree of redshift.

### QD coating and conjugation

QD_9.2_, QD_13.3_, and QD_17.4_ were coated with polyacrylamido(histamine-*co*-TEG-*co*-azido-TEG) (P-IM-N_3_) to generate aqueous azide-functional colloids^13^. Dibenzocyclooctyne (DBCO)-functionalized streptavidin (SAv) was prepared by mixing SAv (AnaSpec) in phosphate buffered saline (PBS; 0.5 mg mL^-1^) with a solution of DBCO-*N*-hydroxysuccinimidyl ester (DBCO-NHS, Click Chemistry Tools) in DMSO (2.5 mM) at a molar ratio of 1:5, followed by repeated pipetting and incubation on ice for 2 h. The conjugate was purified by centrifugal filtration using a filter with 3 kDa molecular weight cutoff (MWCO) at 4 °C. Azide-functional QDs in PBS were then mixed 1:1 with DBCO-SAv (and other ratios for optimization) and allowed to react at room temperature overnight. The reaction was quenched by adding a 50-fold molar excess of 2-azidoacetic acid on ice for 15 min. These conjugates were used directly for 2-step QD-FISH. For 1-step QD-FISH, QD-SAv conjugates were mixed with biotin-labeled probes at a 1:1 molar ratio for 1 h at room temperature.

### QD characterization

Absorption spectra of QD dispersions were acquired using an Agilent Cary 5000 UV–Vis–NIR spectrophotometer. Fluorescence spectra of QD dispersions were collected using a Horiba NanoLog spectrofluorometer, with solutions diluted to eliminate self-quenching. Signal acquisition conditions such as scan time, slit widths, and number of scans were adjusted so that the brightest sample was not saturating the detector (photomultiplier tube) and such that all spectra showed sufficiently high signal-to-noise ratios to yield smooth curves. Transmission electron microscopy images of QDs were obtained using a JEOL 2010 LaB6 high-resolution microscope in the Frederick Seitz Materials Research Laboratory Central Research Facilities at the University of Illinois. Samples were prepared by placing a drop of dilute QD solution in hexane or chloroform on an ultrathin carbon film TEM grid (Ted Pella, #01824) and then wicking the solution off with a tissue. QD-SAv conjugates were characterized by agarose gel electrophoresis (Supplementary Fig. 8) using excess biotin-labeled DNA to confirm SAv conjugation to QDs by a migration shift^13^. The DNA sequence was 5’-Biotin/(T)68 TAGCCA GTG TAT CGC AAT GAC G-3’ (Integrated DNA Technologies). QD-SAv was incubated with biotin-DNA at room temperature for 15 min and electrophoresis was performed in a 2% polyacrylamide, 0.5% agarose gel at 4 °C.

### Cells

HeLa cells (ATCC) were cultured in Eagle’s Minimum Essential Medium (EMEM) with 10% fetal bovine serum (FBS) and 1% penicillin/streptomycin (P/S) at 37 °C in 5% CO_2_. BPH-1 and LNCaP (ATCC) cells were cultured in RPMI Medium 1640 with 10% FBS and 1% P/S at 37 °C in 5% CO_2_. VCaP (ATCC) cells were cultured in Dulbecco’s Modified Eagle’s Medium (DMEM) with 10% FBS and 1% P/S at 37 °C in 5% CO_2_. For FISH studies, cells (1 × 10^5^) were seeded on 18 mm round #1 coverglass in each well of a 12-well cell culture plate and cultured until 70% confluent. The cells were then washed with PBS, fixed with 4% paraformaldehyde for 10 min at room temperature, and permeabilized with 70% (v/v) ethanol for 24 h at 4 °C. For qRT-PCR analysis, BPH-1, VCaP and LNCaP cells (3 × 10^5^) were seeded in 6-well plates and cultured until 80% confluent. For *PTEN* mRNA silencing, cells were transfected with anti-*PTEN* or scrambled siRNA (Santa Cruz) using Lipofectamine 2000 (Thermo Fisher Scientific) following manufacturer protocols at a siRNA concentration of 1.5 fM for 24 h. The medium was then aspirated and the cells were washed with PBS and fixed in 4% paraformaldehyde in PBS at room temperature for 10 min. After two washes with PBS, the cells were permeabilized with 70% (v/v) ethanol for at least 1 h at 2 °C to 8 °C.

### Nucleic acid probes

*GAPDH* mRNA Dye-FISH nucleic acid probes were synthesized and optimized by LGC Biosearch Technologies, labeled with either biotin or CAL Fluor® Red 590 Dye. Probes targeting human *PTEN* mRNA (NM_000314.6) and *A20* mRNA (NM_001270508.1) were designed using Stellaris® Probe Designer (version 4.2, LGC Biosearch Technologies) and are provided in Supplementary Tables 8 and 9.

### Dye-FISH

*GAPDH* mRNA Dye-FISH was performed following standard procedures^8^ using Wash Buffers A and B and Hybridization Buffer supplied by the probe manufacturer. Probe incubation was performed for 16 h in the dark at 37 °C, nuclei were stained with Hoechst 33342 (Thermo Fisher Scientific), and each coverglass was mounted on a slide with 90% glycerol in PBS, sealed using nail polish.

### Two-step QD-FISH

Biotin-labeled FISH probes with the same sequences used for Dye-FISH (LGC Biosearch Technologies) were hybridized with fixed and permeabilized cells on coverglass using identical protocols for Dye-FISH and nuclei were stained with Hoechst 33342. The cells were then blocked for 2 h with blocking conditions as indicated. The optimized mixture contained BSA and DS in 2× saline-sodium citrate (SSC) buffer at pH 7.2. After aspirating the blocking buffer, cells were incubated with 10 nM QD-SAv in 1% (w/v) BSA in 2×SSC buffer at room temperature for 2 h. The ratio between QD:SAv and the time of incubation were independently optimized (Supplementary Fig. 9 and 10). Cells on coverglass were then washed three times with Wash Buffer B before mounting on slides with 90% glycerol in PBS, sealed using nail polish.

### One-step QD-FISH

Fixed and permeabilized cells on coverglass were washed with Wash Buffer A for 5 min and incubated with Hybridization Buffer for 30 min, followed by the optimized blocking buffer from 2-step QD-FISH for 2 h. The cells were then incubated in a mixture of biotin-probe conjugates of QD-SAv (8 nM QD) in 10% formamide, 0.33 mg ml^-1^ yeast RNA, 10 mM ribonucleoside vanadyl complex, 0.1% BSA, and 2XSSC for 16 h in the dark at 37 °C in a sealed humidified chamber. The coverglass was then washed with Wash Buffer A at 37 °C for 30 min and nuclei were stained with Hoechst 33342 for 30 min. The coverglass was then washed with Wash Buffer B for 5 min before mounting on slides with 90% glycerol in PBS, sealed with nail polish.

### Multiplexed QD-FISH

LNCaP cells were transfected with anti-*PTEN* siRNA as described above. *A20* gene expression was induced by treatment with TNF-α (100 ng/mL) in complete medium for 1 h at 37 °C. The cells were then processed following the 1-step QD-FISH procedure and incubated in a mixture of the three QD-SAv conjugates, each conjugated to biotinylated oligonucleotides complementary to mRNA sequences of *GAPDH*, *PTEN*, or *A20*. QD-FISH signals of QD608, QD693 and QD800 were collected using 488 nm laser excitation and 600/37 nm, 698/70 nm or 809/81 nm bandpass emission filters, respectively.

### Imaging

Immediately after preparation, cells were imaged on a Zeiss Axio Observer Z1 inverted microscope with an EC Plan-Neofluar 100$\times$ 1.45 N.A. oil-immersion objective. Images were collected with a Photometrics eXcelon Evolve 512 EMCCD camera controlled through Zeiss Zen software. Hoechst was imaged using 100 W halogen lamp excitation with a 365 nm excitation filter and 445/50 nm emission filter; CAL Fluor® Red 590 Dye was imaged using 561 nm laser excitation and a 600/37 nm bandpass emission filter. QD_13.3_ and commercial QDs were imaged using 488 nm laser excitation and a 600/37 nm bandpass emission filter. QD_17.4_ and QD_9.2_ were imaged using 488 nm laser excitation and a 585 nm long-pass emission filter. Z-stack images of entire cells were collected in 0.22 μm increments. For each sample, 20 areas on the coverglass were selected at random for imaging. To obtain single-molecule fluorescence intensity values, dye-probes and QD-SAv conjugates dispersed in PBS were adsorbed on glass coverslips and imaged via epifluorescence microscopy using identical conditions to those used for FISH images. For each sample, videos during continuous excitation were acquired to identify single molecules by their distinct intensity time-traces using MATLAB algorithms^14^.

### Image analysis

Images were exported as 8-bit uncompressed TIFF files. For 2D image analysis, spot counting in individual cells was performed using the Multiple Target Tracking (MTT) Algorithm based in MATLAB^17^ to determine the location and intensity of each spot. For 3D z-stacks, files were deconvolved using AUTOQUANT X3 (Media Cybernetics, Inc.) and analyzed using IMARIS (Bitplane) for 3D distribution reconstruction and signal spot counting. To ensure that deconvolution did not alter spot numbers, MTT analysis of 2D images was performed before and after deconvolution (Supplementary Fig. 11). In the MTT algorithm, spot detection in images is performed by evaluating each 7 × 7 window in the image using a generalized likelihood ratio test to decide if a spot fits a point spread function, assuming Gaussian noise. Thus the analysis accounts for local background values, rather than global intensities, which is beneficial to account for the nonuniformity of autofluorescence across a cell. The algorithm also subtracts each spot and repeats the analysis until all spots are detected, which is beneficial when a high spatial density of spots is present. The threshold applied for the hypothesis test is normalized as the probability of false positives per 512 $\times$ 512 image (false positives per ~250,000 windows). In Fig. 2, the indicated threshold is the probability of false positive of detected spots in logarithmic scaling, in units of decibels.

### qRT-PCR

Total RNA from cells in 6 well plates was extracted using a RNeasy Mini Kit (QIAGEN) and reverse-transcribed using a High-Capacity cDNA Reverse Transcription Kit (Thermo Fisher Scientific). Primer sequences for human *GAPDH* were 5’-AGG GCT GCT TTT AAC TCT GGT-3’ and 5’-CCC CAC TTG ATT TTG GAG GGA-3’. Primer sequences for human *PTEN* were 5’-CAA GAT GAT GTT TGA AAC TAT TCC AAT G-3’ and 5’-CCT TTA GCT GGC AGA CCA CAA-3’. Primer sequences for human *A20* were 5’-GAC CAT GGC ACA ACT CAT CTC A-3’ and 5’-GTT AGC TTC ATC CAA CTT TGC GGC ATT G-3’^25–28^. All primers were obtained from Integrated DNA Technologies. Real-time qPCR was performed on a Mastercycler® RealPlex2 (Eppendorf).

### Statistical analysis

Data are presented as mean ± s.d. Statistical significance was determined using Student’s *t*-test and analysis of variance (one-way ANOVA) using GraphPad Instat 3 software. After comparing the overall difference between groups, the Tukey’s honestly significant difference (HSD) post-hoc test was used to specify where the differences occurred between groups.

## Electronic supplementary material

Supplementary Information

## Data Availability

The data that support the findings of this study are available from the corresponding author upon reasonable request.
