# Supplementary material for: Enhanced mRNA FISH with compact quantum dots
Source: Nat Commun. 2018 Oct 26;9:4461. doi: 10.1038/s41467-018-06740-x (PMC6203793; doi:10.1038/s41467-018-06740-x)
Supplement: Supplementary file 1 — Supplementary Information [file 41467_2018_6740_MOESM1_ESM.docx]

Supplementary Information

**Enhanced mRNA FISH with Compact Quantum Dots**

Liu *et al.*

**Supplementary Figures**


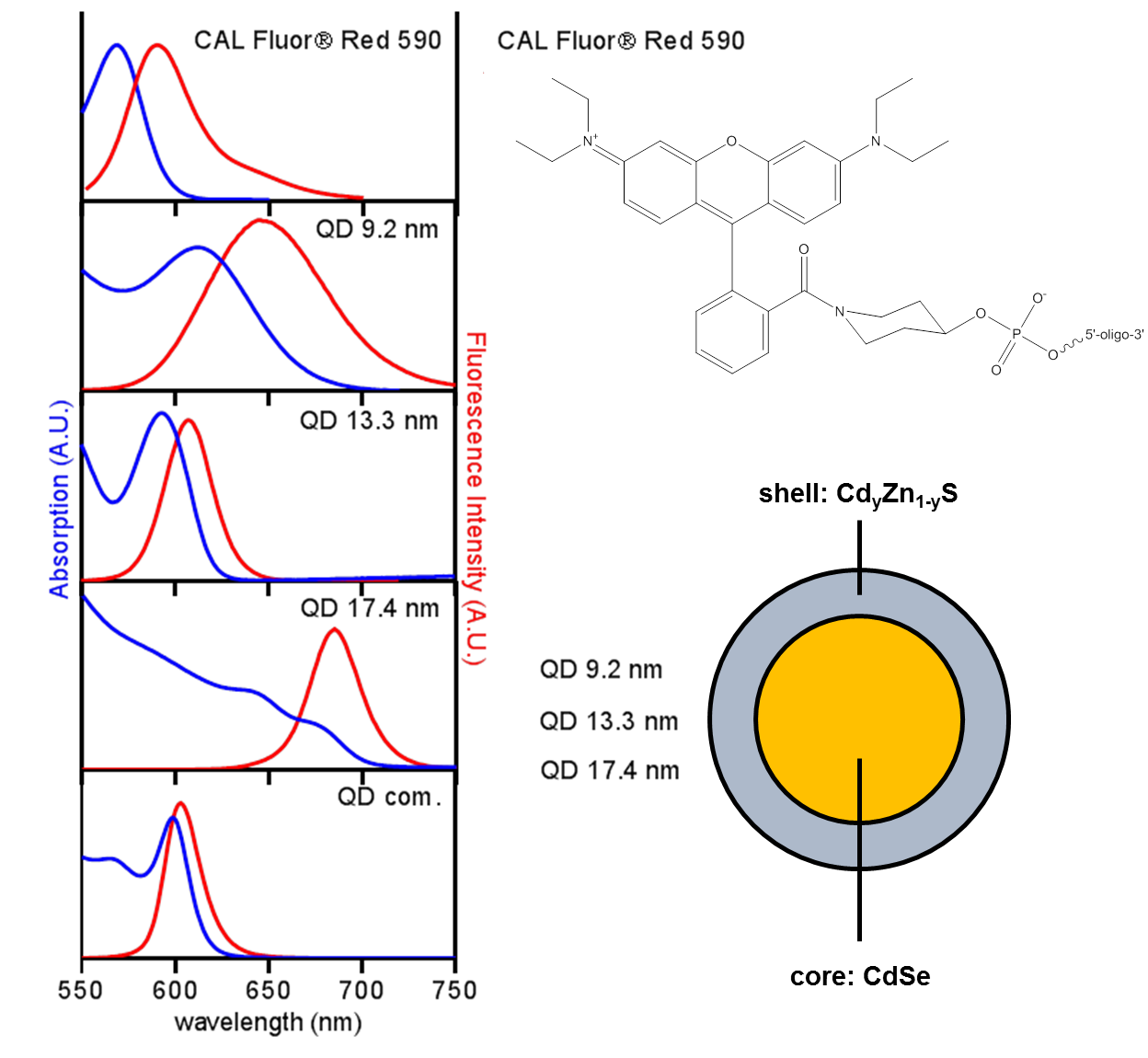


**Supplementary Figure 1**. Spectra and structures of fluorophores used in this work.


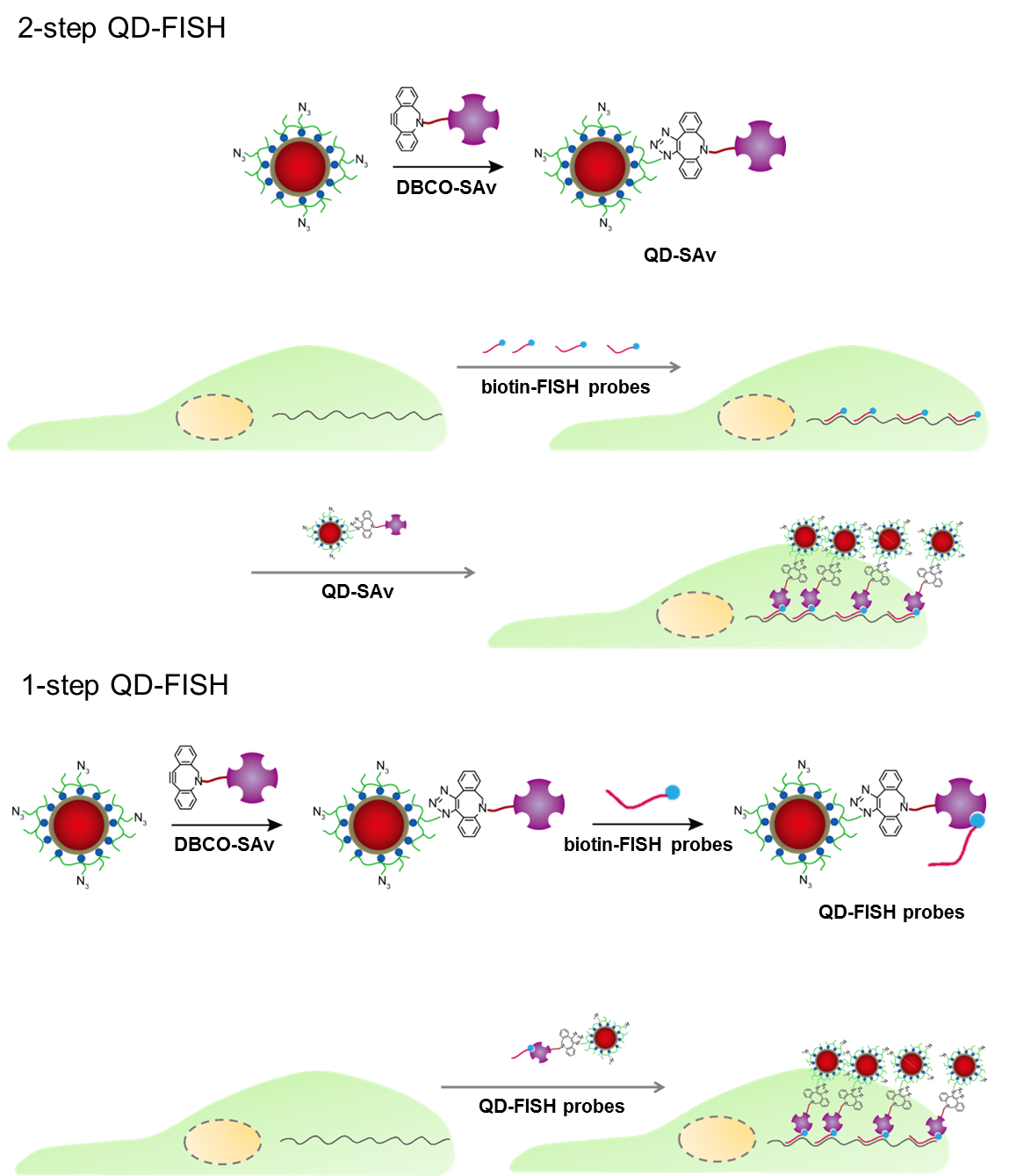


**Supplementary Figure 2**. Schematic depictions of 2-step and 1-step QD-FISH. Azide-functionalized QDs are first conjugated to streptavidin (SAv) functionalized with dibenzylcyclooctyne (DBCO). In 2-step QD-FISH, RNA targets are first hybridized with biotinylated nucleic acid probes and then reacted with QD-SAv. In 1-step QD-FISH, QD-SAv is first conjugated to biotinylated nucleic acid probes and directly hybridized with RNA targets.


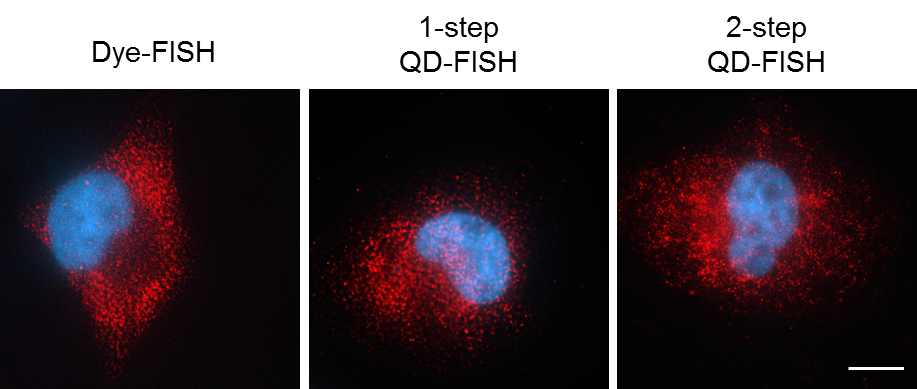


a

**
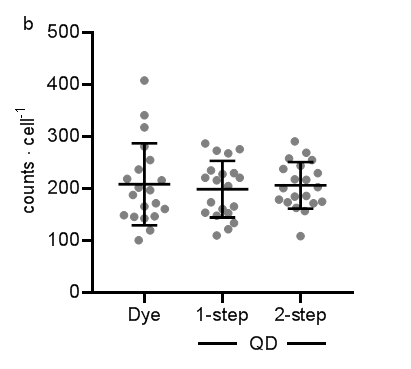
**

**Supplementary Figure 3.** Results from 1-step and 2-step QD-FISH. **(a)** Representative images of HeLa cells stained via FISH for *GAPDH* transcripts using either Dye-FISH, 1-step QD-FISH, or 2-step QD-FISH. Scale bar = 10 μm. **(b)** Scatter plots show that the number of RNA counts per cell is statistically indistinguishable between the three methods (*p* > 0.05; Student’s *t*-test). *N* = 15. Error bars represent s.d.


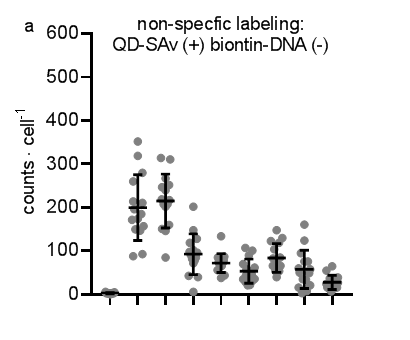


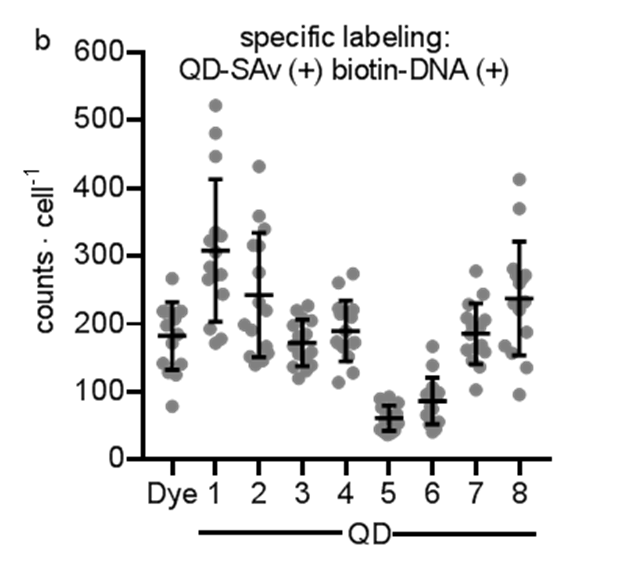


**Supplementary Figure 4.** Optimization of blocking conditions to minimize QD-FISH nonspecific binding, test 1. Treatment conditions include standard conditions for Dye-FISH in addition to colloidal blocking agents (casein and bovine serum albumin, BSA) and anionic blocking agents (poly(acrylic acid), poly(styrenesulfonate), and dextran sulfate) listed below in 2XSSC buffer for 2 hours prior to addition of QD-SAv. For non-specific labeling experiments, samples were not hybridized with biotin-probes. *N* = 15. Error bars represent s.d.

1) 0.1% casein (pH 12.2)

2) 0.1% casein (pH 7.2)

3) 0.1% poly(acrylic acid) (pH 4.75)

4) 0.1% poly(acrylic acid) (pH 7.2)

5) 0.1% poly(styrenesulfonate) (pH 7.2)

6) 0.5% dextran sulfate (pH 7.2)

7) 0.1% dextran sulfate (pH 7.2)

8) 5% BSA (pH 7.2)

**
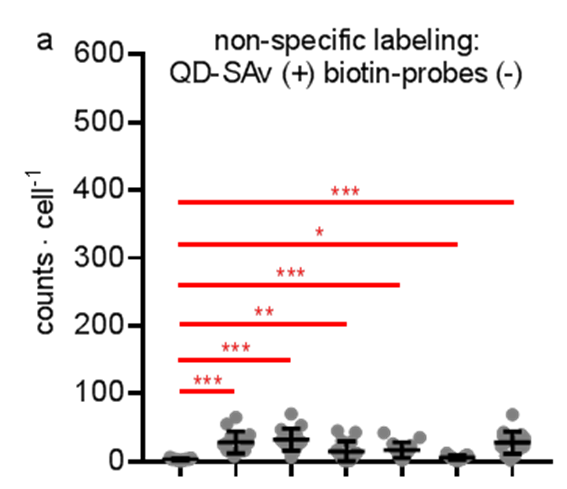
**

**
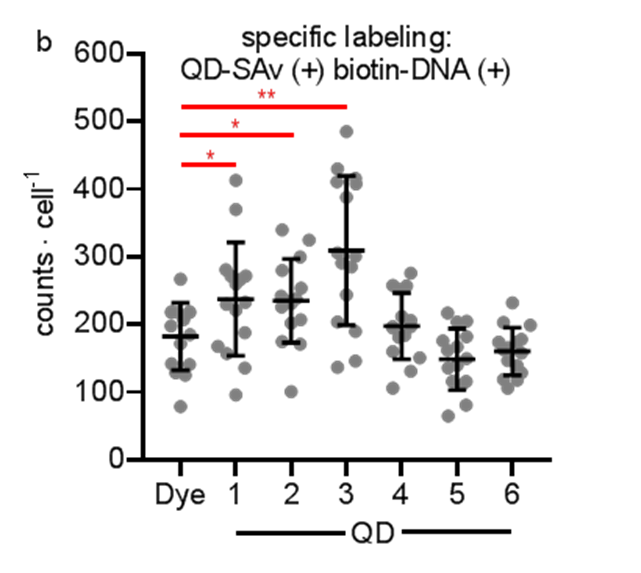
**

**Supplementary Figure 5.** Optimization of blocking conditions to minimize QD-FISH nonspecific binding, test 2. Treatment conditions include standard conditions for Dye-FISH with BSA and additional blocking agents (coating polymer P-IM and dextran sulfate) listed below in 2XSSC buffer for 2 hours either together or in sequence, prior to addition of QD-SAv. For non-specific labeling experiments, samples were not hybridized with FISH biotin-probes. *N* = 15. Error bars represent s.d. * indicates *p* ≤ 0.05; ** indicates *p* ≤ 0.01; *** indicates *p* ≤ 0.001; Student’s *t*-test.

1) 5% BSA

2) 0.1% polyacrylamido(histamine-co-TEG) (P-IM)

3) 5% BSA + 0.1% P-IM

4) 0.1% P-IM, following 5% BSA

5) 0.1% dextran sulfate, following 5% BSA

6) 0.1% dextran sulfate, following 5% BSA + 0.1% P-IM


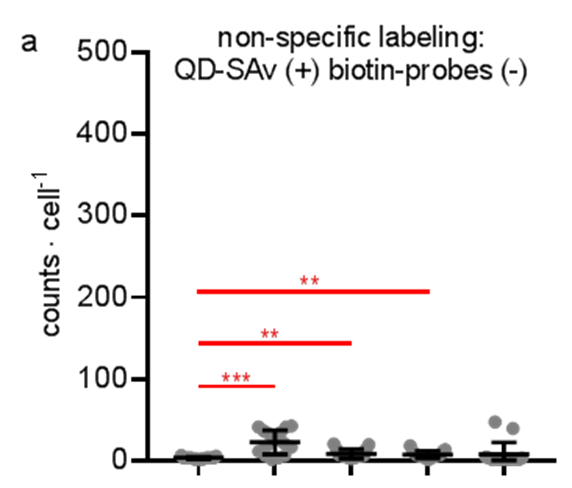


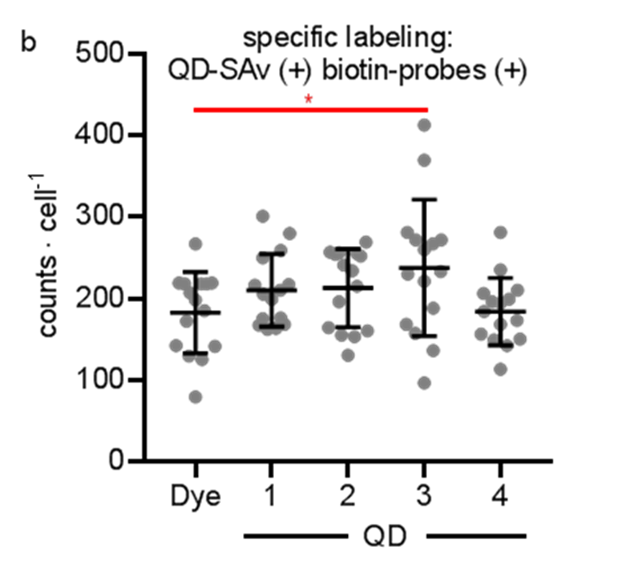


**Supplementary Figure 6.** Optimization of blocking conditions to minimize QD-FISH nonspecific binding, test 3. Treatment conditions include standard conditions for Dye-FISH with human serum albumin (HSA) or BSA, with dextran sulfate in 2XSSC buffer (2 hours), prior to addition of QD-SAv. For non-specific labeling experiments, samples were not hybridized with FISH biotin-probes. *N* = 15. Error bars represent s.d. * indicates *p* ≤ 0.05; ** indicates *p* ≤ 0.01; *** indicates *p* ≤ 0.001; Student’s *t*-test.

1) 5% HSA

2) 5% HSA + 0.025% dextran sulfate

3) 5% BSA

4) 5% BSA + 0.025% dextran sulfate

**Supplementary Fig. 7.** Gel permeation chromatograms of the three QDs used for multiplexed QD-FISH.

**
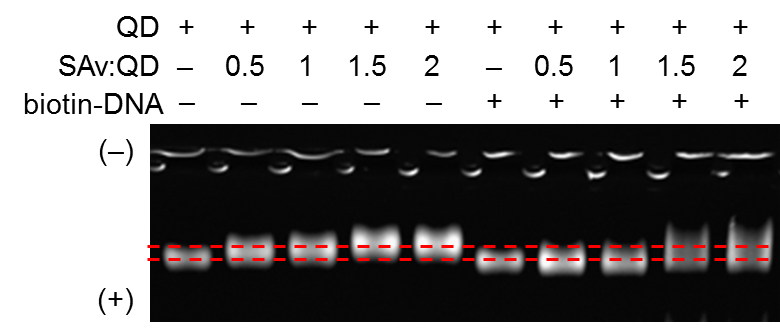
**

**Supplementary Figure 8.** Gel electrophoresis analysis of QD conjugates. QD-SAv conjugations were performed at the indicated molar reaction ratios. Biotin-DNA was added in excess compared with the number of biotin-binding sites on SAv.


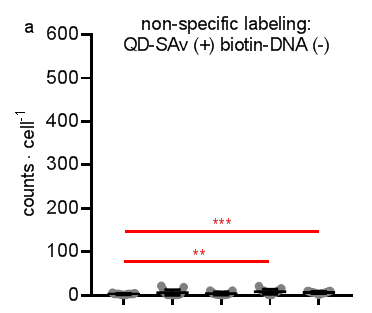


**
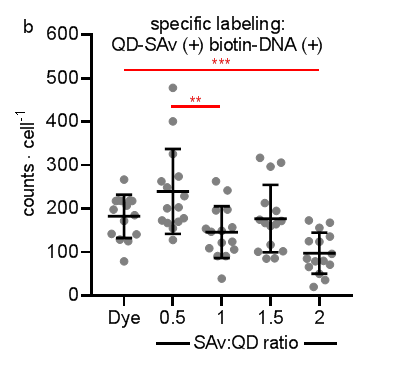
**

**Supplementary Figure 9.** Dependence of QD-FISH results on SAv:QD ratio in HeLa cells. Probes were against *GAPDH* transcripts. The SAv:QD molar reaction ratios were 0.5:1, 1:1, 1.5:1, and 2:1, and were applied using the optimal blocking condition with the 2-step FISH protocol. For non-specific labeling analysis, cells were not hybridized with FISH biotin-probes. *N* = 15. Error bars represent s.d. * indicates *p* ≤ 0.05; ** indicates *p* ≤ 0.01; *** indicates *p* ≤ 0.001; Student’s *t*-test.


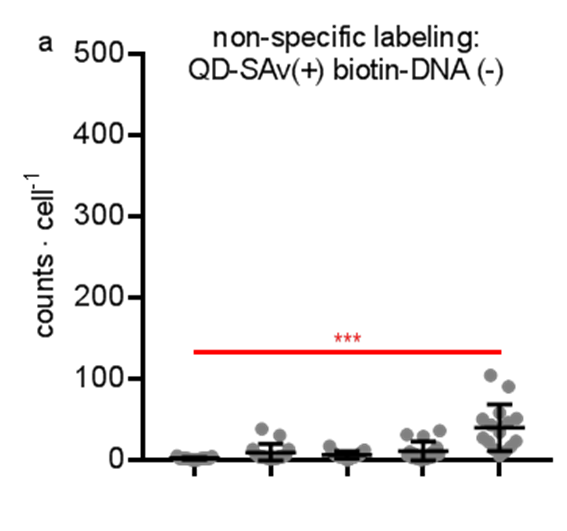


**
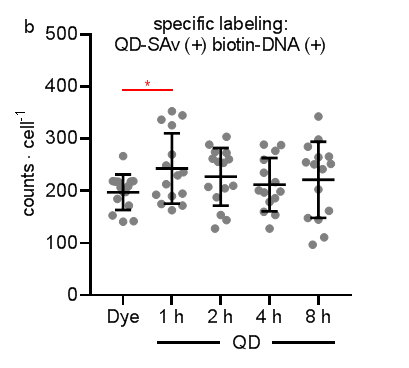
**

**Supplementary Figure 10.** Impact of QD-SAv (1:1 ratio) incubation time for 2-step QD-FISH. The incubation time ranged from 1 h to 8 h using the optimal blocking condition. *N* = 15. Error bars represent s.d. * indicates *p* ≤ 0.05; ** indicates *p* ≤ 0.01; *** indicates *p* ≤ 0.001; Student’s *t*-test.


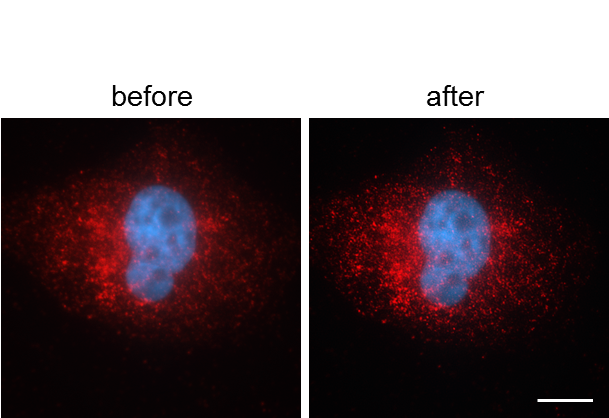
 a

b


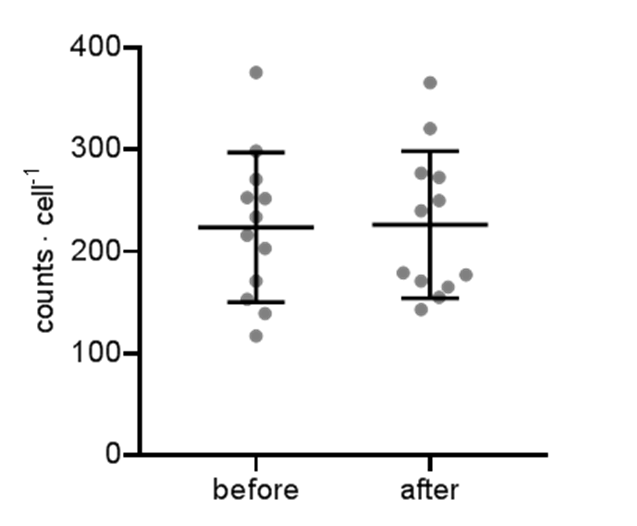


**Supplementary Figure 11.** Comparison between spot counting using QD-FISH images before and after deconvolution. **(a)** Representative images of HeLa cells stained via QD-FISH for *GAPDH*. Scale bar = 10 μm. **(b)** Scatter plots show that the number of RNA counts per cell is statistically indistinguishable before and after deconvolution (*p* > 0.05). *N* = 12. Error bars represent s.d. * indicates *p* ≤ 0.05; ** indicates *p* ≤ 0.01; *** indicates *p* ≤ 0.001; Student’s *t*-test.

**Supplementary Tables**

**Supplementary Table 1.** *p* values for data in Fig. 1b; Student’s *t*-test.

|  | Dye | QD_9.2_ | QD_13.3_ | QD_17.4_ | com. |
| --- | --- | --- | --- | --- | --- |
| Dye |  |  |  |  |  |
| QD_9.2_ | 0.78 |  |  |  |  |
| QD_13.3_ | 0.57 | 0.20 |  |  |  |
| QD_17.4_ | 3.0$\times$10^-6^ | 2.4$\times$10^-6^ | 2.4$\times$10^-6^ |  |  |
| com. | 2.3$\times$10^-4^ | 1.7$\times$10^-5^ | 2.5$\times$10^-5^ | 1.4$\times$10^-3^ |  |

**Supplementary Table 2.** *p* values for data in Fig. 1g; Student’s *t*-test.

|  | **specific labeling** | | | | | | | |
| --- | --- | --- | --- | --- | --- | --- | --- | --- |
| **non-specific labeling** |  | Dye | 1 | 2 | 3 | 4 | 5 | 6 |
|  | Dye |  | 0.038 | 0.83 | 0.010 | 0.47 | 0.37 | 0.031 |
|  | 1 | 2.8$\times$10^-5^ |  | 0.037 | 0.77 | 0.092 | 0.27 | 0.52 |
|  | 2 | 0.032 | 0.028 |  | 0.011 | 0.94 | 0.42 | 0.055 |
|  | 3 | 0.18 | 3.2$\times$10^-5^ | 2.2$\times$10^-4^ |  | 0.083 | 0.31 | 0.73 |
|  | 4 | 0.84 | 6.0$\times$10^-3^ | 1.0$\times$10^-3^ | 0.15 |  | 0.47 | 0.069 |
|  | 5 | 0.024 | 1.0$\times$10^-3^ | 4.1$\times$10^-4^ | 2.0$\times$10^-3^ | 0.84 |  | 0.47 |
|  | 6 | 3.0$\times$10^-3^ | 0.033 | 3.0$\times$10^-3^ | 3.0$\times$10^-3^ | 0.17 | 0.023 |  |

1) 5% BSA (+)

2) 5% BSA (–) + 1‰ DS

3) 5% BSA (+) + 0.125‰ DS

4) 5% BSA (+) + 0.25‰ DS

5) 5% BSA (+) + 0.5‰ DS

6) 5% BSA (+) + 1‰ DS

**Supplementary Table 3.** Optical analysis of FISH spots

|  | *I*_SF_ * | *I*_FISH_ ** | *N* *** |
| --- | --- | --- | --- |
| Dye | 93.0 ± 15.3 | 740 ± 461 | 7.96 |
| QD_13.3_ | 53.8 ± 4.59 | 549 ± 241 | 10.2 |
| QD_17.4_ | 85.7 ± 5.48 | 194 ± 48.4 | 2.26 |

* Average fluorescence intensity of single fluorophore

** Average fluorescence intensity of FISH spot

*** Number of fluorophores per FISH spot

**Supplementary Table 4.** *p* values for data in Fig. 3b; Student’s *t*-test

|  | ctrl | scr/siRNA |
| --- | --- | --- |
| scr/siRNA | 0.12 |  |
| *PTEN*/siRNA | 1.8$\times$10^-6^ | 2.3$\times$10^-6^ |

**Supplementary Table 5.** *p* values for data in Fig. 3c; Student’s *t*-test

|  | scr/siRNA | *PTEN*/siRNA |
| --- | --- | --- |
| ctrl | 0.97 | 3.0$\times$10^-3^ |
| scr/siRNA |  | 0.014 |

**Supplementary Table 6.** *p* values for data in Fig. 3e; Student’s *t*-test

|  | ctrl | scr/siRNA |
| --- | --- | --- |
| scr/siRNA | 0.29 |  |
| PTEN/siRNA | 5.3$\times$10^-6^ | 1.4$\times$10^-8^ |

**Supplementary Table 7.** *p* values for data in Fig. 3f; Student’s *t*-test

|  | scr/siRNA | PTEN/siRNA |
| --- | --- | --- |
| ctrl | 0.88 | 0.015 |
| scr/siRNA |  | 5.0$\times$10^-3^ |

**Supplementary Table 8.** *PTEN* mRNA FISH probe sequences

| 1 | agcattgaaagtttactgca |
| --- | --- |
| 2 | gcagtctgggcatatcaaat |
| 3 | aatagtagttgtactccgct |
| 4 | attaactaggtcagaccaca |
| 5 | gttctgcctaatctatttgc |
| 6 | taatatgcggtgcccgaaat |
| 7 | aaaaccctcattcagacctt |
| 8 | gctgcacatccaaaaagtct |
| 9 | atttgatgctgccggtaaac |
| 10 | ggcaactctgccaaatacta |
| 11 | gtcctttagactattccgaa |
| 12 | caagccagctcatattacaa |
| 13 | ttttcaggtactgtccttac |
| 14 | ccagagtactaccaccaaag |
| 15 | atgttaagcttgtctatgct |
| 16 | tgcacattaggacatgaggg |
| 17 | tggaagtctagcactcttga |
| 18 | acacacatcaagatgctact |
| 19 | ctctaaccattataagcaca |
| 20 | gctcaaagatggcagtttac |
| 21 | aagttggctccaacatttca |
| 22 | tagctacacttaagccattt |
| 23 | gctatttattcaactaccct |
| 24 | ggcaaacatgttcaagagga |
| 25 | aaggacttgggatggtacag |
| 26 | cctccatgttacatattgga |
| 27 | aactctataaatgctgctct |
| 28 | ggtcctattcaatctgtatt |
| 29 | gtgaacaatttcagcaccaa |
| 30 | gggggagcactatgaagaaa |

**Supplementary Table 9.** *A20* mRNA FISH probe sequences

| 1 | ATACAAAGCCTGAGGAAGGA |
| --- | --- |
| 2 | ACAGCTTTCCGCATATTGCT |
| 3 | GTGTATCGGTGCATGGTTTT |
| 4 | GGCAAGTTCTGAACATTTCC |
| 5 | TCCCGAAACTGAGGACAAAA |
| 6 | TTTCTGTCGATGAGGGCTTT |
| 7 | AGTTTCTTCTGGCTTTCCAG |
| 8 | TACTGAGAAGTGGCATGCAT |
| 9 | TACCAAGTCTGTGTCCTGAA |
| 10 | TTGCGTGTGTCTGTTTCCTT |
| 11 | CAGTTGCCAGCGGAATTTAA |
| 12 | CCCGTTTCAACAAATTCCTG |
| 13 | CAGTTCCGAGTATCATAGCA |
| 14 | GTCTGTGGAAGCCATTTTGA |
| 15 | TTGTACTGAAGTCCACTTCG |
| 16 | GAGGATGTTGCAAAGGACAA |
| 17 | GGAACCTGATTCCAAACTTC |
| 18 | ATTCCACCCACTTTCAAAGG |
| 19 | ATGGGGTATCTGTAGCATTC |
| 20 | AAATGATGGCTGTCATAGCC |
| 21 | AAGTGGAACAGCTCGGATTT |
| 22 | AAGTCTTCAAATCTTCCCCG |
| 23 | CGGGGATTTCTATCACCATT |
| 24 | ATGAGATGAGTTGTGCCATG |
| 25 | TTCATCCAACTTTGCGGCAT |
| 26 | GCCATTTCTTGTACTCATGC |
| 27 | TTCACAGACATGAAGAAGGG |
| 28 | TGAGCACTCATGGCATAAAG |
| 29 | TTTGATTCTTTTGCCGCCTC |
| 30 | GAGTTCAGCTTTGGGAGTTT |
| 31 | TCACTGAACAGAAAAGGGCT |
| 32 | TGCTGCACATTCAGTGTGAA |
| 33 | GCAACGTTCACAAAATCCGT |
| 34 | AATGTCCTGGTAACATCCTG |
| 35 | TGAAGCAAGTACTGCAGATC |
| 36 | TCTGGAGTCCCAAAATACAC |
| 37 | AAACACAGTGTGCAAAAGCC |
| 38 | TCAAACATGGTGCTTCCAAG |
| 39 | GAAACACTTCTGGCAGTATC |
| 40 | GGCCTCATGAAATCTCTGAT |
| 41 | TCAGTTGCTCTTCTGTCCTT |
| 42 | TTGAGGTGCTTTGTGTGGTT |
| 43 | TTGCCAAAATGATCACAGGC |
| 44 | AAAGCATTCGTTGCAGTAGC |
